# PET/CT Evaluation of Brain Aβ and Metabolism in a Marmoset Model of Alzheimer's Disease Based on Tau Seeding

**DOI:** 10.1002/alz70856_107737

**Published:** 2026-01-10

**Authors:** Leila Letica, Diego Szczupak, Lauren Bailey, Seung‐Kwon Ha, Yongshan Mou, Lauren R Dubberley, Ryan Robert Dyer, Bei Zhang, Thais Rafael Guimaraes, Lauren K Hayrynen Schaeffer, Emily S. Rothwell, Jung Eun Park, Sang‐Ho Choi, David J Schaeffer, Amantha Thathiah, Stacey J Sukoff Rizzo, Afonso C Silva

**Affiliations:** ^1^ University of Pittsburgh School of Medicine, Pittsburgh, PA, USA

## Abstract

**Background:**

Alzheimer's disease (AD) is the leading form of dementia, affecting 7 million Americans over age 65. AD is characterized by the accumulation of amyloid‐β (Aβ) plaques in the brain and the formation of neurofibrillary tangles (NFT) of hyperphosphorylated tau. Aβ and tau accumulate in the brain decades before cognitive decline. However, the relationship between Aβ and tau remains unclear. We hypothesized that injecting tau into the entorhinal cortex and hippocampus of aging marmosets would trigger NFT formation and propagation, accelerating impairments across various AD‐related sensory, motor, cognitive, and non‐cognitive phenotypes associated with disease progression.

**Method:**

Ten marmosets aged 65‐142 months (5F) received bilateral stereotactic injections of AAV‐P301LTau into the entorhinal cortex and hippocampus. A neuroimaging series consisting of anatomical and functional MRI along with PET/CT, using the radioligands ^11^C‐PiB to track Aβ and ^18^F‐FDG to monitor glucose metabolism, was employed at a pre‐seeding baseline and again at 6 and 12 months post‐seeding. The marmosets were fasted for two hours before the scans, and ^11^C‐PiB and ^18^F‐FDG were injected IV at an average dose of 18.5 MBq. Dynamic datasets were analyzed for standard uptake values (SUV) using PMOD v3.4. Statistical tests were conducted using GraphPad Prism 9. Cognitive, behavioral, and social assessments were conducted. Blood‐based biomarkers (Aβ40, Aβ42, GFAP, NFL, and total Tau) were measured using ELISA. In deceased marmosets (*N* = 3, 2F), immunohistochemistry for total Tau, 3RTau, 4RTau, and Thr231 was performed.

**Result:**

Whole brain ^11^C‐PiB uptake increased, and ^18^F‐FDG decreased between pre‐seeding and post‐seeding scans. Age‐ and sex‐matched non‐seeded marmosets also showed lower ^11^C‐PiB uptake than seeded marmosets, suggesting a rise in Aβ following tau seeding. Blood‐based biomarkers indicated an initial post‐seeding spike in total Tau and a return to baseline levels. IHC analysis confirmed the successful seeding and propagation of tau from the entorhinal cortex and hippocampus to the prefrontal and posterior regions of the brain cerebellum.

**Conclusion:**

We established, characterized, and validated a marmoset model of sporadic AD based on seeding tau to accelerate the emergence of AD‐related phenotypes. These marmosets will be invaluable in translational research to elucidate the pathogenic mechanisms of AD and contribute to novel therapeutics for AD.

